# Ubiquitin-Mediated Degradation of Aurora Kinases

**DOI:** 10.3389/fonc.2015.00307

**Published:** 2016-01-18

**Authors:** Catherine Lindon, Rhys Grant, Mingwei Min

**Affiliations:** ^1^Department of Pharmacology, University of Cambridge, Cambridge, UK; ^2^Department of Cell Biology, Harvard Medical School, Boston, MA, USA

**Keywords:** Aurora kinase, AURKA, AURKB, ubiquitin-mediated proteolysis, mitosis, APC/C

## Abstract

The Aurora kinases are essential regulators of mitosis in eukaryotes. In somatic cell divisions of higher eukaryotes, the paralogs Aurora kinase A (AurA) and Aurora kinase B (AurB) play non-overlapping roles that depend on their distinct spatiotemporal activities. These mitotic roles of Aurora kinases depend on their interactions with different partners that direct them to different mitotic destinations and different substrates: AurB is a component of the chromosome passenger complex that orchestrates the tasks of chromosome segregation and cytokinesis, while AurA has many known binding partners and mitotic roles, including a well-characterized interaction with TPX2 that mediates its role in mitotic spindle assembly. Beyond the spatial control conferred by different binding partners, Aurora kinases are subject to temporal control of their activation and inactivation. Ubiquitin-mediated proteolysis is a critical route to irreversible inactivation of these kinases, which must occur for ordered transition from mitosis back to interphase. Both AurA and AurB undergo targeted proteolysis after anaphase onset as substrates of the anaphase-promoting complex/cyclosome (APC/C) ubiquitin ligase, even while they continue to regulate steps during mitotic exit. Temporal control of Aurora kinase destruction ensures that AurB remains active at the midbody during cytokinesis long after AurA activity has been largely eliminated from the cell. Differential destruction of Aurora kinases is achieved despite the fact that they are targeted at the same time and by the same ubiquitin ligase, making these substrates an interesting case study for investigating molecular determinants of ubiquitin-mediated proteolysis in higher eukaryotes. The prevalence of Aurora overexpression in cancers and their potential as therapeutic targets add importance to the task of understanding the molecular determinants of Aurora kinase stability. Here, we review what is known about ubiquitin-mediated targeting of these critical mitotic regulators and discuss the different factors that contribute to proteolytic control of Aurora kinase activity in the cell.

## Introduction

**Figure 1:**
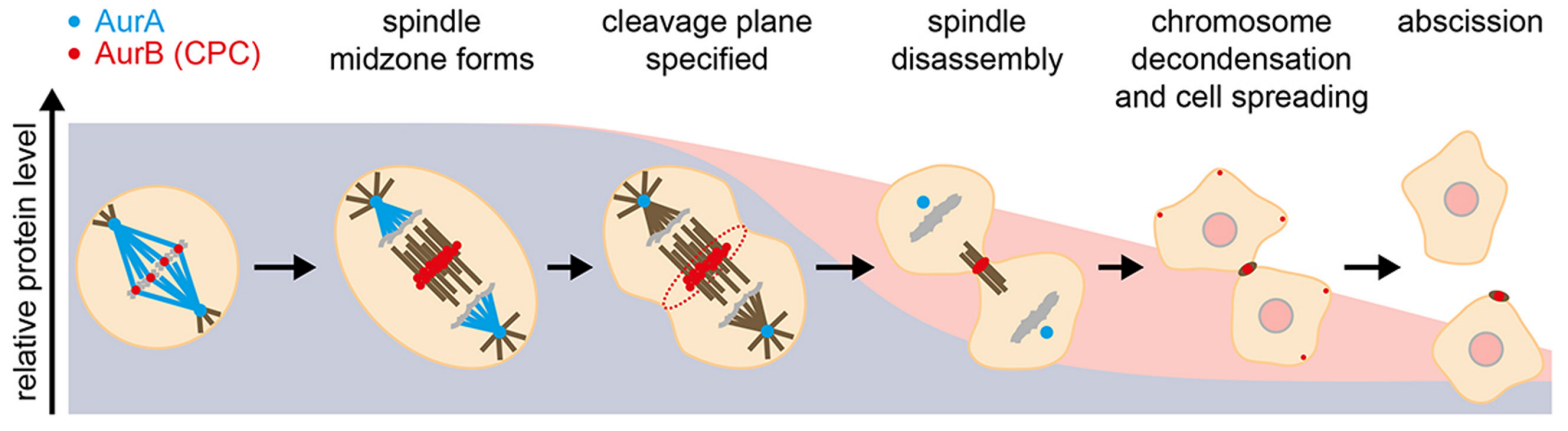
**Events during mitotic exit that are influenced by Aurora kinases, illustrated against their degradation profiles**. Blue, AurA; red, AurB.

Aurora kinases are critical regulators of eukaryotic cell division. Their structure, activities, and functions have been extensively reviewed elsewhere (1–4) and will be mentioned only briefly here. Although Aurora kinases share a high degree of homology in their kinase domains, they play distinct roles in cell division (Figure 1). Aurora A (AurA) is an upstream element in the cascade of kinase activities that control progression from G2 to M phase (through Bora-mediated activation of Plk1) and further plays direct roles in the maturation of the centrosome, in microtubule (MT) nucleation, and in the activation of other components required to build a bipolar mitotic spindle. AurA has a large number of substrates and interactors and alternative modes of activation, with different partners thought to give rise to distinct pools of active kinase. Aurora B (AurB), on the other hand, resides as an obligatory component of the chromosome passenger complex (CPC; along with INCENP, survivin, and borealin), which is essential for chromosome condensation and organization during mitosis, including a critical role as an effector of the mitotic checkpoint in regulating kinetochore-MT attachments on the mitotic spindle. Both Auroras have a predicted disordered N-terminus. This disordered region is more extensive in AurA and is found to mediate much of the specificity in AurA interactions, including those required for its functions at the centrosome (5).

It is striking that a version of AurA bearing a single-point mutation that switches its major mitotic interaction from TPX2 to INCENP can rescue knockdown of AurB (6, 7), and consistent with this observation, the two kinases appear to have many shared substrates. Other, specific, AurA or AurB substrates are likely to be constrained in their specificity in a cellular context through colocalization with one or other of the Auroras (8–11). Perhaps not surprisingly then, some lower eukaryotes were found to have a single Aurora kinase that carries out roles at both centrosomal and chromosomal locations and which can functionally substitute for either AurA or AurB in mammalian cells (12, 13). Spatial organization of Aurora kinase activity is thus thought to have arisen through the acquisition of different binding partners. The divergence of AurA and AurB functions in higher eukaryotes presents an interesting paradigm of differential regulation of kinase activity at specific subcellular domains. One of the elements contributing to such spatiotemporal regulation is differential targeted proteolysis.

The discovery of the ubiquitin–proteasome system (UPS) for targeted destruction of proteins (proteolysis) provided the framework for understanding how mitotic exit is driven by the activity of a multisubunit ubiquitin ligase complex known as the anaphase-promoting complex/cyclosome (APC/C) (14, 15). Targeting of securin and mitotic cyclins by the APC/C is necessary and sufficient for chromosome segregation and mitotic exit, respectively. Two decades of research on the APC/C have elucidated many features of its action and identified a large number of additional targets, which include the Aurora kinases. How, and why, the APC/C targets many different substrates with high temporal specificity remains an intriguing question in mitosis control. A resetting of the protein landscape of the cell must occur in preparation for interphase, for example, to rid the cell of factors that contributed to the assembly of the mitotic spindle. In some cases, however, it has been shown that the destruction of specific substrates contributes to the orderly progression of mitotic exit (16–19). Aurora kinases are two such substrates whose targeting by the APC/C and its coactivator Cdh1 contributes to the correct dosing, timing, and localization of their activities (17, 19).

There is now a substantial body of literature pointing to additional, non-mitotic roles of AurA, indicating a requirement for regulating Aurora kinase activity in interphase. It seems likely that a substantial fraction of AurA is protected from APC/C–Cdh1 activity in G1 phase, since APC/C–Cdh1 activity is predominantly nuclear (20–22) and AurA largely cytoplasmic (in contrast to AurB, which is strongly localized to the nucleus). Therefore, alternative UPS pathways may regulate cytoplasmic AurA outside of mitosis, and a number of candidate UPS components are reported in the literature.

The importance of regulating Aurora kinase activity is well established. In this review, we will consider the importance of proteolysis for the activity of Aurora kinases in mitosis and in interphase and what is known about mechanisms of Aurora kinase proteolysis. A bias in our review toward AurA reflects the fact that far more is known about proteolysis of this Aurora paralog in higher eukaryotes.

## Why Are Aurora Kinases Targeted for Proteolysis?

### Spatiotemporal Organization of Aurora Kinase Activity Through the Cell Cycle

Proteolytic pathways have been shown to effect dosage compensation to enforce stoichiometric expression of the components of multiprotein complexes (23), and indeed, both AurA and AurB are destabilized by the loss of respective interaction partners TPX2 and INCENP (24, 25). This observation may be widely applicable to proteins, such as Aurora kinases, containing short linear interaction motifs (SLiMs) (26) within extended unstructured regions. SLiMs can adopt specific structures upon interaction. Various pools of AurA act through different interactors, generating structures with distinct autophosphorylation profiles (for example, interaction with nucleophosmin generates a phospho-Ser89 epitope in an active pool of AurA distinct from that activated by phosphorylation in the T-loop at Thr288) (27). Destabilization could be a default mode to constrain Aurora kinase activity unless protected by interaction, helping to maintain distinct, spatially defined pools of AurA and AurB activities.

We note that during early mitosis, the APC/C is proposed to play a role in “dosing” spindle-associated factors by eliminating components in excess of those required for the assembly of the correctly sized bipolar mitotic spindle (28). In this model, binding to MTs directly stabilizes proteins, such as HURP, which are otherwise turned over rapidly by the APC/C. We speculate that such default targeting by the APC/C could be a characteristic of mitotic regulators that assists their clearance from the cell as the machinery of cell division disassembles at the end of mitosis.

### Execution of Mitotic Exit

Aurora kinases play critical roles in orchestrating events at mitotic exit (Figure 1). Elucidating them has been a challenging task, given the multiple functions of the Auroras earlier in mitosis. In recent years, however, the use of chemical genetics and development of specific small molecule inhibitors have helped decrypt roles of Aurora kinases after anaphase onset. Activity of either AurA or AurB is essential for disassembly of the metaphase spindle (29). Furthermore, AurA activity is required for anaphase spindle dynamics and central spindle formation, with AurA inhibition reducing anaphase pole-to-pole separation, resulting in a disorganized midzone with sparse MTs. Although the exact molecular mechanism remains to be elucidated, TACC3 and the dynactin subunit p150Glued have been identified as AurA substrates mediating anaphase spindle elongation (27, 30, 31). AurB plays critical roles during anaphase as the CPC relocates to the midzone of the anaphase spindle and from thence to the equatorial cortex, where AurB activity is essential for furrowing (32, 33). At the completion of cytokinesis, AurB is found on MTs flanking the midbody, where it retains activity to control the timing of abscission through the CHMP4C component of the ESCRTIII complex (34–37).

Downregulating Aurora kinase activity is also important for mitotic exit. The activation of counteracting phosphatases (38) does not appear sufficient to reverse the functional phosphorylation events mediated by the Auroras, since non-degradable versions perturb the organization of mitotic exit. The gain-of-function phenotypes exhibited by non-degraded Auroras could point to kinase-independent roles, but more likely mean that the kinases retain some activity when dephosphorylated in the activation loop (39). We propose therefore that targeted Aurora degradation, as a tool for tuning the activity of the kinases, is a critical element of their functions in mitotic exit.

In Cdh1 knockdown, consequently, anaphase spindle organization is perturbed, and the spindle over-elongated, in a fashion that can be rescued by codepletion of AurA and mimicked by expression of non-degradable AurA (17). AurA, p150, and TACC3 may act to translate the precise downregulation of AurA into remodeling of the anaphase MT network. Cleavage furrow ingression occurs earlier in Cdh1 knockdown than in control cells and is accompanied by the premature appearance of AurB at the equatorial cortex (19). Whether this effect is mediated through stabilization of AurB, through disruption of the central spindle caused by stabilization of AurA, or through a different Cdh1 substrate, is not known. The effect of Cdh1 knockdown on abscission timing has not been reported, but stabilization of AurB is likely to delay this process, as well as contributing to the genomic instability reported in Cdh1^−/−^ MEFs (40).

### Establishing Interphase

**Figure 2:**
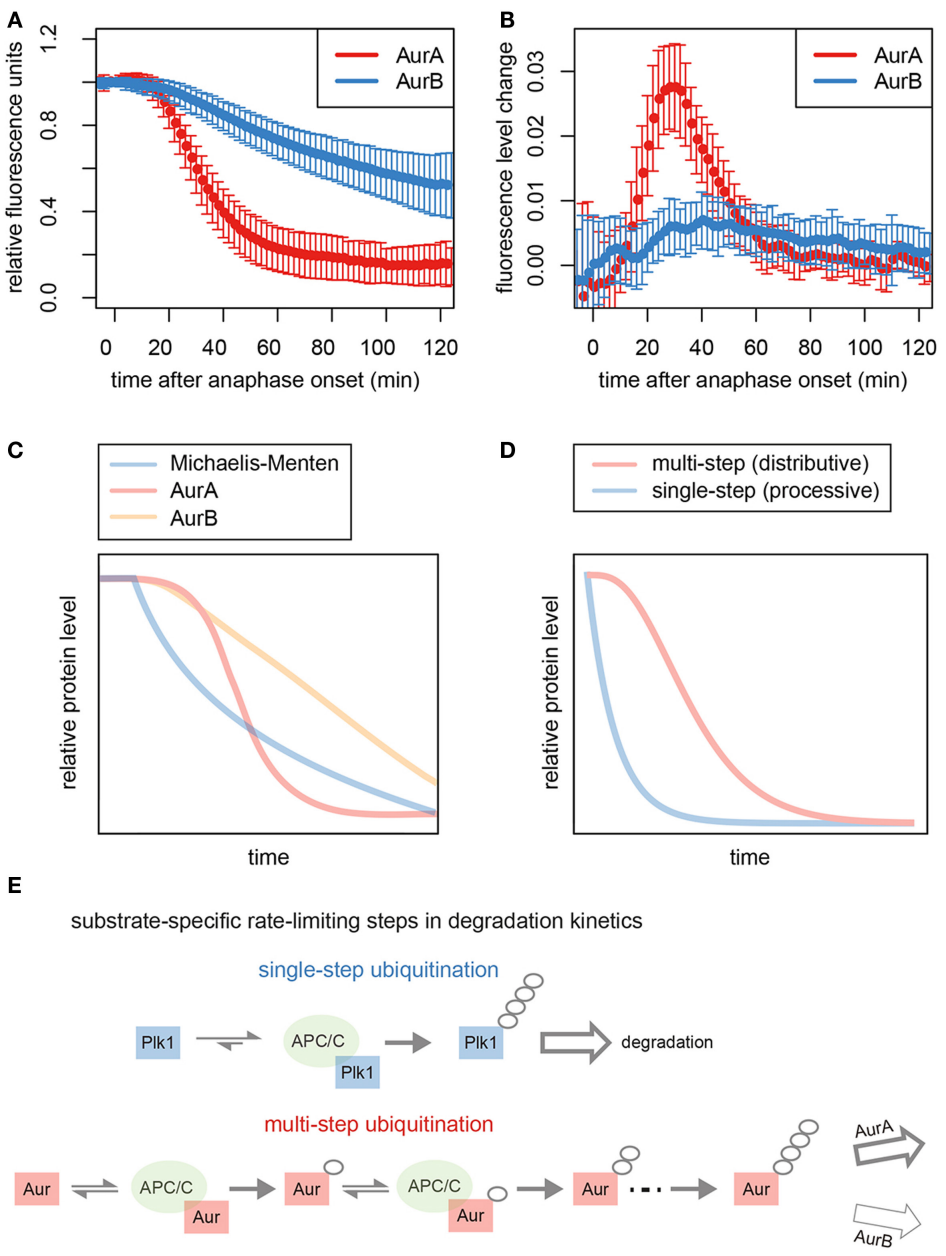
**Kinetics of Aurora kinase destruction during mitotic exit**. **(A)** Total levels of Venus-tagged AurA and AurB in cells passing through mitosis. Data taken from Min et al. (47). Fluorescence measurements from single cells were used to generate averaged progress curves for the degradation of each substrate (*n* ≥ 50). **(B)** Plots of the changing rate of degradation over time for the averaged progress curves show that the maximum rate of AurA degradation is fivefold higher than that of AurB. **(C)** Simulation of first-order (Michaelis–Menten) kinetics predicts a theoretical degradation curve showing an exponential decrease in substrate levels over time that resembles the degradation curves that we have previously described for other substrates of the APC/C, such as Plk1, RacGAP1, and KIFC1 (16, 48), and is consistent with the idea that for these substrates, ubiquitination is the single rate-limiting step for proteolysis (since the rate of proteolysis depends on the amount of substrate present). **(D)** Modeling of distributive ubiquitination of a substrate, where a threshold number of stepwise ubiquitin modifications is required to generate the product that can be processed for proteasomal degradation, compared to a processive ubiquitination process. The simulated reaction exhibits the sigmoidal/switch-like response that characterizes degradation of AurA. **(E)** Schematic to explain kinetics of degradation of different substrates. Processive ubiquitination of substrates, such as Plk1, is achieved by a single binding event to the APC/C, and substrates rate limited by single-step ubiquitination are degraded with first-order kinetics. By contrast, Aurora kinases bind to the APC/C multiple times to acquire polyubiquitin chains. AurA, rate-limited by this multistep ubiquitination, exhibits switch-like degradation kinetics. Degradation of AurB is likely governed by a post-ubiquitination step.

Several mitotic processes that depend on Aurora activity must be reversed as cells return to interphase. Reorganization of the cell cytoskeleton requires degradation of AurA for disassembly of spindle poles and of AurB for formin-mediated cell spreading (17, 19). For other processes, such as AurA-mediated mitochondrial fissioning (41), the role of Aurora degradation has not yet been established. Ubiquitination of AurB is proposed to be required for its p97-dependent extraction from chromatin to allow chromosome decondensation and nuclear envelope formation (42). More generally, tuning of AurB activity may tie the timing of abscission to the state of the nucleus at the passage to interphase: AurB activity has been proposed to delay abscission in response to delays in nuclear pore assembly (43), and recent studies show that the same ESCRT machinery regulated by AurB in the process of abscission is involved in resealing the nuclear envelope at the start of interphase (44, 45). A gradient of AurB activity emanating from the midzone is proposed to coordinate these events with sister chromatid separation in a checkpoint-like manner (46). What has become increasingly apparent is that AurB acts as both sensor and effector in the transition from mitosis to interphase, with its activity carefully modulated through localization, exposure to phosphatases, and degradation. This role for AurB provides a rationale for the very different degradation kinetics of AurA and AurB observed at the end of mitosis (Figure 2).

### Regulating Cell Fate

A substantial body of literature points to additional, non-mitotic roles of Aurora kinases. AurA is required for reabsorption of the primary cilium in serum-stimulated quiescent cells and for migration of postmitotic neurons during development (49, 50). Both AurA and AurB are implicated in cell fate decisions, AurA through effects on stability of N-myc and p53, GSK3 signaling, and Notch pathways (51–55) and AurB through modulating the epigenetic states of histone H3, for example, in maintaining the differentiated state of C2C12 myoblasts and in transient transcriptional reprograming of events in interphase nuclei (56–58). As already shown for the role of AurA in postmitotic neurons, the activities of Aurora kinases in each of these processes could be regulated by proteolysis (50).

### Proteostasis and Cancer

The systematic overexpression of AurA in cancers was noted early on after the discovery of Aurora kinases (59, 60) and is now recognized as an important driver of many cancer types, often as a result of amplification of the AurA gene, located on the 20q amplicon (for example, the most common amplicon in colorectal cancer) (61, 62). AurA is thought to contribute to chromosome instability (CIN) during mitosis through its effects on MT dynamics (63), raising the possibility that control of AurA levels is required to protect cells from CIN (64). The functions of Aurora kinases in interphase could also contribute to the tumorigenic nature of AurA overexpression – perhaps more efficiently than functions in promoting chromosome segregation in mitosis. Notably, kinase-independent roles, such as the protein–protein interaction between AurA and MYCN protein that stabilizes MYCN in neuroblastoma (53), provide a link between regulation of AurA levels and proliferation. Drugs that disrupt the AurA–MYCN interaction may offer a therapeutic route to treating neuroblastoma (65).

To what extent, then, is pathological expression of AurA a problem with regulation of protein stability? One model for conditional AurA overexpression showed that *in vivo* overexpression of mouse AurA from a transgene did not result in increased AurA protein levels, since these were suppressed by proteolysis under physiological conditions (66). A recent proteogenomic survey of colorectal cancer reported that, in general, mRNA overexpression driven by gene amplifications was not reflected in overexpression at the protein level, suggesting that the latter is buffered by posttranscriptional regulation (62). Therefore, overexpression of AurA protein in cancers may indicate changes in the stability of the protein, either changes in AurA or in the pathways that regulate it. For example, stabilization of AurA through constitutive phosphorylation of a critical residue, Ser51, has been reported in head and neck cancers (67). Coexpression of TPX2 may be another route to stabilizing AurA in cancers, contributing to excess AurA activity after 20q amplification, since *AURKA* and *TPX2* are both located on the long arm of chromosome 20 (68).

In the following sections of this review, we will discuss factors that determine, or influence, the ubiquitin-mediated regulation of Aurora kinase levels in the cell.

## The Kinetics of AurA and AurB Degradation at Mitotic Exit

In mammalian cells, anaphase substrates of the APC/C fall into groups that show distinct kinetics of degradation when measured in single cell assays *in vivo*. These kinetics are determined by the multilayered complexity of the UPS, which includes posttranslational modifications (PTMs) on substrates (and on the APC/C) and other characteristics of substrate interactions with the APC/C that determine the on-rate and the residence time of the substrate (69). The activity of deubiquitinating enzymes (DUBs) and of other ubiquitin modifiers can also influence the degradation of ubiquitinated substrates, and the p97 AAA-ATPase may be required to unfold ubiquitinated substrates to render them accessible for degradation (70). The topology of ubiquitinated substrates undoubtedly influences their interaction with the proteasome, since polyubiquitin chains and an unstructured region that serves as the degradation initiation region need to be in the right proximity to one another for proteasomal proteolysis (71).

*In vivo* assays of GFP-tagged Aurora kinases report on the timing and kinetics of their degradation, which begins 10 min after anaphase onset (Figure 2A) (47). The timing of degradation onset for AurA and AurB is identical by this assay. However, their rates of degradation are very different (Figure 2B), explaining the long-standing observation that AurA is removed from the cell well ahead of AurB during mitotic exit (16, 17, 72, 73).

Progress curves for substrate degradation can provide information on the kinetics of the underlying reactions. The progress curve of GFP Venus-tagged AurA is consistent with the idea that distributive (stepwise) ubiquitination of AurA determines the kinetics of disappearance of this substrate (Figures 2A,C,D). AurA was previously shown to be a distributive substrate *in vitro*, where building a proteolytic ubiquitin chain requires multiple rounds of substrate–APC/C binding each binding event considered an independent and reversible step (69, 74) (Figure 2E). In contrast to the switch-like kinetics of AurA-Venus degradation, AurB-Venus degradation progressed at a rate that was slow but constant – even when AurB-Venus levels were low (Figures 2A–C) – with the inference that degradation is governed by a rate-limiting step with low catalytic activity and high affinity of the rate-limiting enzyme for the substrate (75). Since both substrates appear ubiquitinated to the same extent during mitotic exit (47, 76), we propose that this rate-limiting step in AurB degradation occurs post-ubiquitination. We note that both of these progress curves are distinct from those of other substrates we have studied, such as Plk1 and KIFC1 (16, 48), which show first-order (or Michaelis–Menten) kinetics (rate of disappearance dependent on substrate concentration) (Figures 2C,D). First-order kinetics is consistent with a model where processive (single-step) substrate ubiquitination would be rate limiting for degradation. It seems therefore that distinct steps in processing of the Aurora kinases underlie their characteristic degradation curves and differential removal from the cell (Figure 2E).

## How Are Aurora Kinases Targeted in Mitotic Exit?

### Aurora Kinases Are Cdh1-Dependent Substrates of the APC/C

AurA was found early on to be an efficiently degraded substrate of the APC/C (77). Its efficient degradation in *in vitro* assays using extracts from human cells or *Xenopus* oocytes has facilitated identification of substrate-specific determinants of degradation (78–81). The APC/C relies on either of the two coactivators, WD40 repeat factors Cdc20 or Cdh1 (FZR1 in humans). AurA is specifically targeted by Cdh1 *in vitro* (79, 80, 82) and is robustly stabilized by depletion of Cdh1 in various systems (17, 40, 72). Recombinant AurB is not degraded efficiently in the same *in vitro* assays (78), but AurB levels are highly sensitive to Cdh1 in cell-based assays (17, 47, 72, 83).

The specificity of the APC/C for its substrates shifts as cells pass through mitosis. As cells enter anaphase, specificity switches from a relatively restricted pool of substrates to a large one that may number in the hundreds (76, 84). Although the switch from Cdc20 to Cdh1 was originally thought to account for this change in specificity (85), it is now evident that a majority of substrates are efficiently degraded during mitotic exit in the absence of Cdh1, through altered targeting specificity of APC/C–Cdc20 (17, 47, 72, 73, 86). For these substrates, therefore, the requirement for Cdh1 only reveals itself in G1 phase, or in *in vitro* assays, when the APC/C is in a dephosphorylated state that cannot interact with Cdc20. The strict dependence of AurA and AurB targeting on Cdh1, even when APC/C–Cdc20 is active, marks them out from other APC/C substrates. Only Cdc6 is known to share this specificity (86), and we propose that the shared timing of destruction of these substrates signals the moment of activation of APC/C–Cdh1 in mitotic exit.

**Figure 3:**
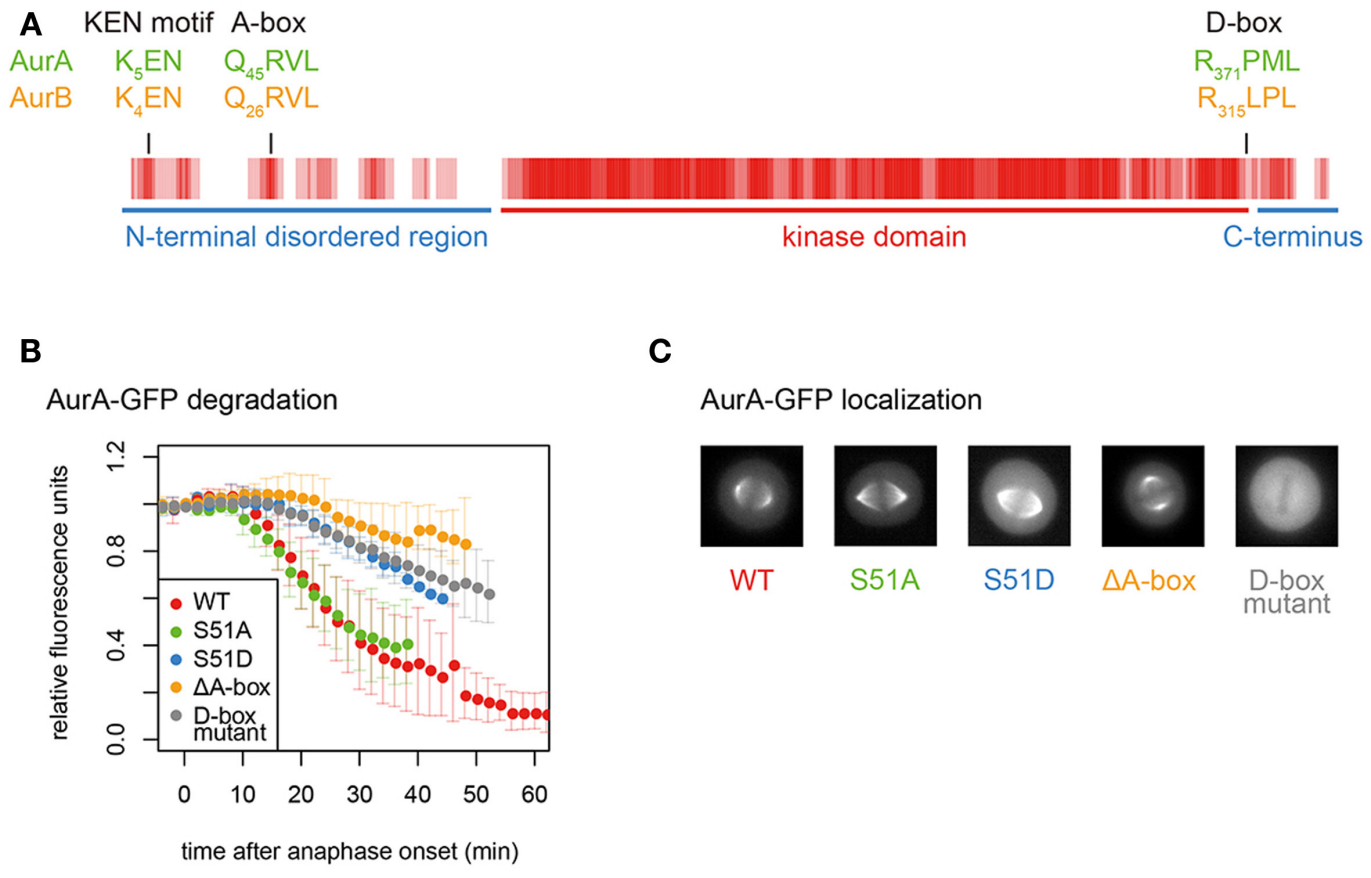
**Conserved degrons in Aurora kinases**. **(A)** Aurora kinase degrons are the most conserved motifs outside the kinase domain. The sequence alignment of AurA and AurB was converted to a vector of values corresponding to conservation at each position (from 4 for fully conserved position to 0 for no conservation). Rolling averages of a five-residue window across the whole alignment is presented as a heat map. Therefore, the shade of red indicates residue conservation between the two paralogs. **(B)** Degradation plots for A-box- (including S51-) and D-box-mutated versions of AurA-GFP, as described in Ref. (81). Fluorescence levels measured over time in single cells exiting mitosis are normalized to anaphase onset. ΔA-box = Δ31–66; D-box mutant = R371A, L374A. **(C)** Mitotic localization of mutants analyzed in **(B)**, showing loss of functional localization of the D-box mutant.

The unique specificity of Aurora kinases and Cdc6 is probably determined by the way in which they interact with the APC/C. It is well known that APC/C substrates contain receptor motifs, the so-called degrons, which are recognized and bound by coactivator-associated APC/C (87, 88). Now, structural studies [recently reviewed in Ref. (89)] are able to show the direct binding of coactivators, via their WD40-repeat propellors, to canonical degrons in substrates. However, although Aurora kinases contain such canonical degron motifs (D-boxes and KEN motifs), it is not clear what roles these play, since an additional non-canonical degron, called the “A-box” is also present (79) (Figure 3).

### Aurora Kinases Contain Multiple Degrons

#### Canonical Degrons

APC/C substrates are usually characterized by the presence of a D-box (“destruction box,” consensus RxxL), first identified in the N-terminus of B-type cyclins (90). However, the D-box alone may not be sufficient for productive binding of most substrates: APC/C–substrate interactions are more likely governed by the weak interactions through multiple degrons categorized as SLiMs (91, 92). The most important of these additional degrons is the KEN motif, first identified in Cdc20, which lacks a D-box (88). The KEN motif binds to a different surface of the coactivator WD40 domain to the D-box, and is prevalent in APC/C substrates (and in 8% of the proteome). Aurora kinases contain conserved D-boxes and a KEN motif (Figure 3A). *In vitro* degradation assays identify the functional D-box of Aurora kinases as that conserved in a position close to the C-terminal end of the kinase domain (79, 81–83, 93). However, the orientation of the RxxL within the known structure of the kinase domain (94, 95) raises a question mark over how it could be accessible to the APC/C. Mutation of this motif not only abrogates the destruction of GFP-tagged AurA in cells undergoing mitotic exit but also abolishes the localization of AurA to any mitotic structures, rendering *in vivo* assessment of its role problematic (Figures 3B,C).

It is notable that all mitotic interactions of Aurora kinases are acutely sensitive to disruption in the C-terminal region (96, 97). Structural simulations of AurA–TPX2 interaction predict that the cancer-associated somatic mutation S155R in AurA, which prevents interaction with TPX2 (97), increases disorder in the C-terminus (98). Therefore, interaction with binding partners through the C-terminus maintains the overall structure of the kinases. Loss of critical interactions could allow partial unfolding of Aurora kinases prior to targeting of the D-box by the APC/C, explaining the destabilization seen after loss of TPX2 or INCENP (24, 25).

An alternative idea, where the D-box is not assumed to function as a degron, is that the C-terminus of Aurora kinase is required for an intramolecular interaction, such as that proposed for AurA (99), influencing the structure of the N-terminus. The structure of the N-terminus could, in turn, determine the availability of SLiM-type degrons in the N-terminus for targeting by the ubiquitination machinery.

Among these SLiMs are the Aurora KEN close to the N-terminus and the “A-box.” Despite multiple reports from *in vitro* studies that KEN plays no role in AurA mitotic destruction (79, 80, 82, 93), it contributes to the degradation in cell-based assays of both AurA and AurB (19, 100, 101). However, the AurA K5 within the KEN motif is ubiquitinated during mitotic exit (101), in apparent conflict with function as a degron. Structural or cross-linking studies of KEN interactions will be required to resolve the function of this motif. The A-box appears to qualify as a degron since the A-box-deleted version of AurA localizes correctly in mitosis and is resistant to mitotic exit degradation (Figures 3B,C).

#### The A-Box, a Specific Determinant of Aurora Kinase Destruction

The A-box motif was identified in AurA (residues 31–66) as the sequence required for APC/C–Cdh1-mediated destruction of AurA in mitotic/G1 extracts (79). More recent studies indicate that Q_45_RVL – conserved in AurB – is sufficient to mediate degron function in both kinases (67, 81, 100).

The A-box is predicted to mediate an atypical degron interaction with APC/C–Cdh1, but the structural basis for this specificity has not been investigated. To our knowledge, the only structurally defined contributor of specificity for Cdh1 is the “A-motif” found in APC/C inhibitor Acm1 in yeast (91, 102). Distinct from the Aurora A-box, the “A-motif” is a 10 amino acid loop, including a key FxLxYE region that interacts with a non-canonical binding site on Cdh1 via a salt bridge and two hydrophobic interactions. Aurora kinases may employ an equivalent strategy in assembling substrate-specific APC/C–coactivator–E2 complexes, as discussed below.

### Aurora Kinases Are Ube2S-Dependent Substrates of the APC/C

The APC/C ubiquitinates its targets in conjunction with two E2 enzymes, Ube2C (UbcH10) and Ube2S. Ube2C adds the first, or “priming,” ubiquitin, and can generate short chains on substrates, while Ube2S elongates ubiquitin chains through the addition of K11-specific ubiquitin linkages (103, 104).

K48 linkages are the canonical proteasomal degradation signal, while K11 linkages have recently been found to mediate rapid degradation of mitotic substrates (47, 105, 106). The two E2s bind non-competitively to the APC/C (UbcH10 via the RING domain subunit APC11 and Ube2S via APC2), acting together as a highly efficient module for rapid targeting of substrates to the proteasome. The coactivators Cdc20 and Cdh1, as well as participating in substrate recognition, also promote the activity of APC/C through a critical substrate-induced stabilization of E2 binding to the APC/C (92, 107, 108).

**Figure 4:**
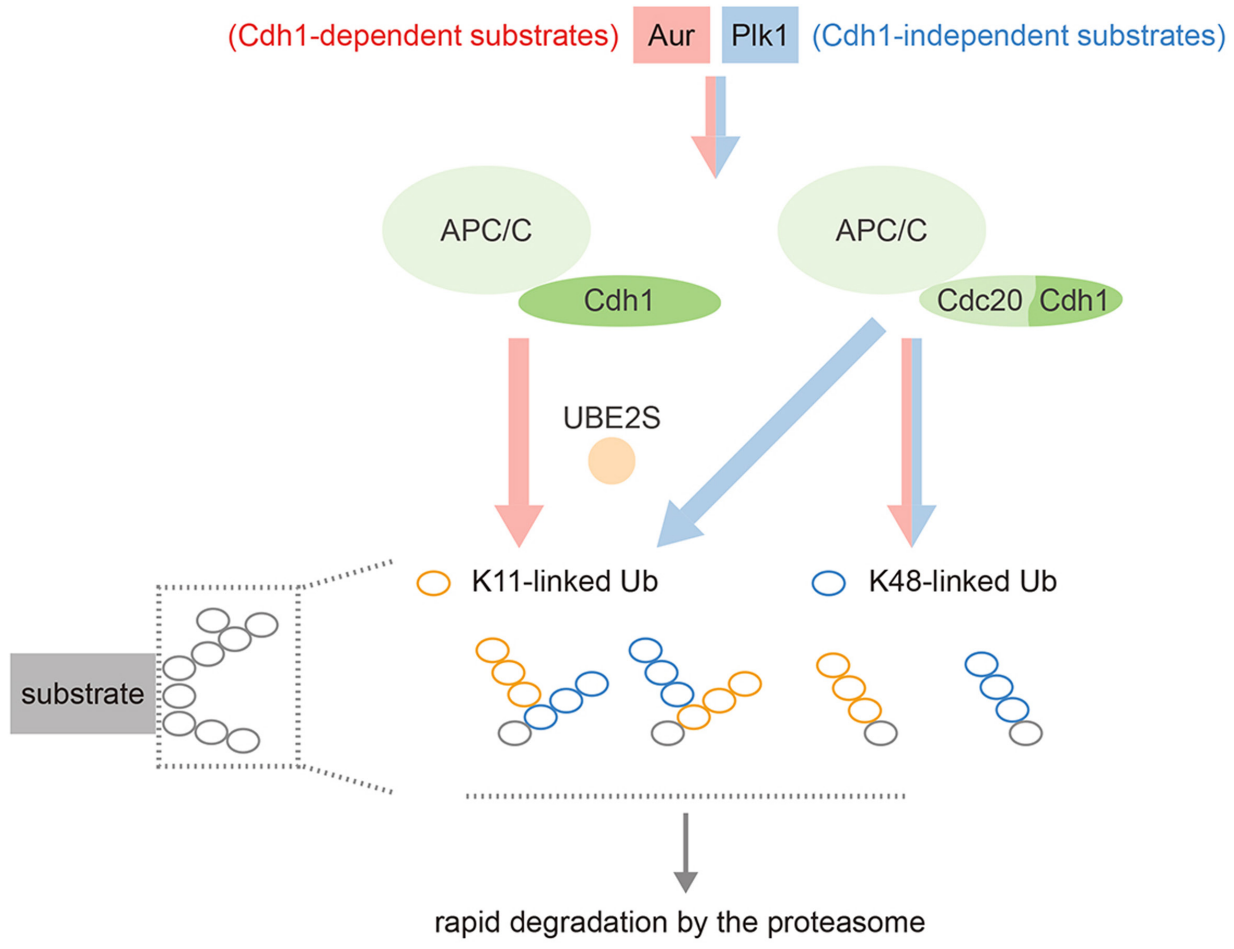
**Schematic illustrating the complexity of APC/C substrate specificity after anaphase onset**. Most substrates can be targeted by either APC/C–Cdc20 or APC/C–Cdh1, working with both Ube2C and Ube2S (blue stream). Cdh1-only substrates, although they can still bind Cdc20, show restricted targeting by the APC/C–Ube2S that depends on Cdh1 (red stream). APC/C–Ube2S is required to generate the K11 linkages for rapid degradation at the proteasome.

Our own work has shown that Aurora kinases are decorated with a mixture of K48- and K11-linked ubiquitin chains during mitotic exit, and that Ube2S is essential both for the K11 linkages and for efficient degradation of these substrates (47). Other substrates are able to receive K11 chains in the absence of Cdh1 (presumably via Cdc20–Ube2S), while in Cdh1-depleted cells, Aurora kinases lose all their K11 chains but are still ubiquitinated with K48 chains in an APC/C-dependent manner, presumably because Ube2C recruitment can still occur (47). In other words, Aurora degradation depends on Cdh1 not for recruitment to the APC/C, but for generating K11 linkages via Ube2S (Figure 4).

### Why Cdh1 Specificity?

The functional significance – if there is any – of exclusive targeting by Cdh1 is not clear. Cdh1 could be specifying the timing of Aurora kinase destruction with respect to anaphase functions. It has been shown that APC/C–Cdh1 assembly depends on prior anaphase Plk1 destruction (109), thus the exclusive targeting of Aurora kinases by Cdh1 imposes strict order on the destruction of these substrates; Plk1 ahead of AurA (16). In the case of the replication factor Cdc6, which shows identical coactivator specificity and degradation timing to AurA, it is suggested that delayed degradation with respect to the licensing inhibitor geminin, a substrate of APC/C–Cdc20, creates a short but clearly defined window of opportunity for replication licensing during mitosis (86). Similarly, there may be an event in mitotic exit that requires Aurora kinase activity in the absence of Plk1 or some other Cdc20 substrate.

An alternative explanation for the Cdh1 specificity is suggested by the progress curve of AurA degradation (Figure 2) (47). The “switch-like” kinetics imparts robustness to the destruction of this substrate once a cell is committed to mitotic exit (activation of Cdh1) and may depend on low processivity arising from weak Cdh1-substrate interactions. Finally, specificity for Cdh1 may introduce possibilities for modulating the degradation of substrates via chain editing – for example, the DUB USP37 interacts with APC/C–Cdh1 to modulate K11 linkages on at least one substrate (110).

## Regulation of Aurora Kinase Degradation

### Posttranslational Modification of Aurora Kinases

Recent advances in proteomics have not only started to reveal the identity of *in vivo* ubiquitination sites (111), but also the complexity of PTMs that can modify the fate of target proteins. Tens of thousands of ubiquitinated lysines are known, although mostly these lack functional annotations. We find it interesting that only four endogenously ubiquitinated sites have been found for AurA but a large number for AurB (18 out of 22 lysines). Many of these ubiquitination sites are likely to serve non-proteolytic functions, by creating or disrupting interfaces with other partners. For example, CUL3-KHLH9/13/21-dependent ubiquitination is required for the correct localization of AurB in anaphase (112, 113). We have found it necessary to disrupt multiple lysines in the N-terminus of AurB to significantly disrupt AurB degradation in mitotic exit (Mingwei Min, Catherine Lindon, unpublished data), suggesting that several or all of these ubiquitinated lysines could carry chains that contribute to processing of AurB at the proteasome. The same is not true for AurA, which seems to rely strongly on its most N-terminal lysine, K5 (101) for mitotic exit degradation. This difference in lysine usage could explain the differential degradation kinetics that we measure for the two substrates, for example, if removal of ubiquitin chains at the proteasome prior to proteolysis is slow, as has been suggested (114).

Lysines can be subject to several PTMs beyond ubiquitination. Functionally important sumoylation of both Aurora kinases has been reported to occur on conserved lysines that are also reportedly ubiquitinated (in humans, AurA K258 and AurB K202), but it is not known how this might have impact on potential ubiquitination at these sites (115–117). The deacetylase SIRT2 has been found to strongly regulate both AurA and AurB levels *in vivo* (proposed to explain the high rate of tumorigenesis in SIRT2^−/−^ mice) (118). Investigation of the underlying mechanism was unable to detect acetylation on AurA lysine residues, but found that acetylation of APC/C coactivators interferes with their function. Aurora-specific recruitment of SIRT2 could therefore act to promote Aurora degradation, either directly or indirectly. We note that recent work showing acetylation on ubiquitin as a potential route to switches in chain specificity, or between mono- and poly-ubiquitinated states, could put deacetylases center stage as regulators of protein degradation dynamics (119).

Finally, proteomics approaches have identified several functional phosphorylation sites on AurA (most of them autophosphorylation sites), reviewed recently elsewhere (4). The most interesting from the point of view of Aurora stability is phosphorylation on the serine immediately downstream of the QRVL motif (S51 in human AurA), which appears to regulate the degron function of the A-box since phosphomimic mutation of this residue (S51D) stabilizes AurA in mitotic exit as efficiently as removal of the A-box (67, 79, 81, 120) (Figure 3B). This PTM has therefore been proposed to control the degradation of AurA at the end of mitosis (79, 81, 120). However, replacement of S51 with a non-phosphorylatable residue (S51A) does not alter the timing of degradation of AurA during mitotic exit (Figure 3B). Dephosphorylation on this residue would therefore be a permissive state, rather than the trigger of AurA destruction.

The serine residue S4, adjacent to the mitotic exit-specific ubiquitin acceptor lysine K5, is also phosphorylated *in vivo* (121) and phosphomimic replacement increases ubiquitination efficiency on the neighboring lysine (101).

### Interactors of Aurora Kinases

AurA has multiple interactors, many of which, like TPX2 (25), are reported to modulate AurA levels through ubiquitin-dependent and -independent pathways. There is limited information about modulators of AurB stability, probably reflecting that AurB levels are effectively suppressed in interphase. Examples of interactors that influence AurA stability are Nedd9/HEF1, Pleckstrin-homology-like domain protein PHDLA1, PUM2, LIMK2, and FAF1 (122–126), and in many cases, this stabilization occurs through competition for access to regions of AurA usually targeted by the UPS.

However, the switch-like function of the pS51 PTM in stabilizing AurA may provide another route to modifying AurA turnover: recent studies by Erica Golemis and colleagues show that AurA can be activated through Ca^2+^-induced binding of Calmodulin (CaM) to the A-box region, and that CaM binding depends on the presence of serine residues, including S51, that are phosphorylated under the same conditions (127, 128). CaM binding to pS51 can be predicted therefore to stabilize AurA by blocking access to the A-box region. In this model of AurA regulation, autophosphorylation of AurA on S51 connects activity and stability and allows for functionally relevant stabilization of active forms of the kinase through Ca^2+^-mediated signaling. There are reports that CaM also binds to AurB (127, 129), although in this case, it is proposed that AurB is stabilized through competition for FBXL2 access to a region that does not include the A-box (129).

## Other UPS Components Targeting Aurora Kinase

Although the APC/C appears to be the major E3 regulating Aurora kinase levels *in vivo* and destroys most of the detectable Aurora kinase in cells that exit mitosis, a small pool of AurA is thought to remain to fulfill interphase functions, either protected from APC/C-mediated destruction (perhaps through activity of a DUB, or through sequestering in the cytoplasm) or a newly synthesized pool as cells return to interphase. So, are there UPS components that turn over Aurora kinases in interphase cells? Candidate E3 ubiquitin ligases are CHFR, shown to target AurA both *in vitro* and *in vivo* (130), the BRCA1-associated BARD1 that interacts with AurB (131), and SCF complexes containing a number of reported F-box proteins. FBXW7, FBXL7, and FBXL2 are all reported to target Aurora kinases (53, 132–135), but it is not clear how well *in vitro* targeting predicts *in vivo* pathways, especially since the effects of overexpressing or depleting F-box proteins on global levels of Aurora kinases are frequently rather modest. It seems likely that small subpopulations are being targeted in each case (for example, FBXL7 localizes to the centrosomes), as part of the complex spatial organization of kinase activity that underlies the multiple and divergent functions of these kinases. Interestingly though, dramatic stabilization of AurA is seen after treatment of cells with GSK3B inhibitor (136). In this study, GSK3B was proposed to promote FBXW7 targeting of AurA through priming a phospho-degron located in the kinase domain. However, phosphorylation of AurA by GSK3B on S283/4 is known to promote autophosphorylation on S342 that is inhibitory to AurA activity (137), making it likely that GSK3B can govern AurA stability indirectly through conformational effects.

Dramatic effects on AurA levels are also reported in response to a factor called AURKAIP1, an AurA-interacting protein that promotes AurA destruction in a ubiquitin-independent manner. AURKAIP1 may direct AurA to the proteasome through an interaction that competes with the ubiquitination machinery, since polyubiquitination is abolished upon overexpression of AURKAIP1 (138). However, AURKAIP1 turns out to be a mitochondrial ribosomal protein (139), such that the physiological relevance of these observations remains to be demonstrated. Finally, another interesting study reported that AurA is a substrate for the ubiquitin conjugating enzyme Ube2N, with which it interacts directly through an N-terminal domain that requires the residue F31. An F31I polymorphism that has lost Ube2N interaction is preferentially amplified in tumors (140), raising the still unanswered question of whether increased stability of AurA F31I could explain its role in colon cancer susceptibility.

## Conclusion

Aurora activity is a major regulator of the cell cycle, with a separation of functions between paralogous Aurora kinases whose degradation kinetics in vertebrates have apparently evolved hand in hand with their specialization. The distribution of functions of the ancestral Aurora between two more specialized paralogs is a process that phylogenies indicate to have occurred more than once in eukaryotic lineages (12): Aurora kinases A and B in vertebrates, and the two Aurora kinases in flies and worms, arose via independent duplication events, following similar pathways toward specification of function. Such convergent evolution of Auroras suggests positive selection for specialization of two pools of Aurora kinase. While the kinase activity of the paralogs remains conserved, the regulatory modules including short linear motifs in their disordered regions, have largely diverged. We suggest this could be linked to the importance of differentially regulating pools of Aurora kinase activity in time. Although not well studied in other species, differential proteolysis is a feature of human Aurora kinases that strictly depends on these divergent terminal regions. While different interactors can achieve spatial regulation of Aurora kinase activity, differential proteolysis adds complexity to the control of Aurora kinase activity in a temporal domain.

## Author Contributions

All authors contributed to writing the review and preparing figures.

## Conflict of Interest Statement

The authors declare that the research was conducted in the absence of any commercial or financial relationships that could be construed as a potential conflict of interest.
